# Earlier Initiation of Antiretroviral Treatment Coincides With an Initial Control of the HIV-1 Sub-Subtype F1 Outbreak Among Men-Having-Sex-With-Men in Flanders, Belgium

**DOI:** 10.3389/fmicb.2019.00613

**Published:** 2019-03-26

**Authors:** Lore Vinken, Katrien Fransen, Lize Cuypers, Ivailo Alexiev, Claudia Balotta, Laurent Debaisieux, Carole Seguin-Devaux, Sergio García Ribas, Perpétua Gomes, Francesca Incardona, Rolf Kaiser, Jean Ruelle, Murat Sayan, Simona Paraschiv, Roger Paredes, Martine Peeters, Anders Sönnerborg, Ellen Vancutsem, Anne-Mieke Vandamme, Sigi Van den Wijngaert, Marc Van Ranst, Chris Verhofstede, Tanja Stadler, Philippe Lemey, Kristel Van Laethem

**Affiliations:** ^1^Laboratory of Clinical and Epidemiological Virology, Department of Microbiology and Immunology, Rega Institute for Medical Research, KU Leuven, Leuven, Belgium; ^2^AIDS Reference Laboratory, Department of Clinical Sciences, Institute of Tropical Medicine Antwerp, Antwerp, Belgium; ^3^National Reference Confirmatory Laboratory of HIV, National Center of Infectious and Parasitic Diseases, Sofia, Bulgaria; ^4^Infectious Diseases and Immunopathology Section, ‘L. Sacco’ Department of Biomedical and Clinical Sciences, ‘L. Sacco’ Hospital, University of Milan, Milan, Italy; ^5^AIDS Reference Laboratory, CUB-Hopital Erasme, Université Libre de Bruxelles, Brussels, Belgium; ^6^Laboratory of Retrovirology, Department of Infection and Immunity, Luxembourg Institute of Health, Esch-sur-Alzette, Luxembourg; ^7^Serviço de Patologia Clínica, Laboratorio de Biologia Molecular, LMCBM, Centro Hospitalar Lisboa Ocidental, Hospital Egas Moniz, Lisbon, Portugal; ^8^Centro de Investigação Interdisciplinar Egas Moniz, Instituto Universitário Egas Moniz, Almada, Portugal; ^9^EuResist Network GEIE, Rome, Italy; ^10^Institute of Virology, University of Cologne, Cologne, Germany; ^11^Unit of Medical Microbiology, Institute of Experimental and Clinical Research, Université catholique de Louvain, Brussels, Belgium; ^12^PCR Unit, Clinical Laboratory, Kocaeli University, İzmit, Turkey; ^13^Research Center of Experimental Health Sciences, Near East University, Nicosia, Cyprus; ^14^Molecular Diagnostics Laboratory, National Institute for Infectious Diseases ‘Matei Bals’, Bucharest, Romania; ^15^IrsiCaixa AIDS Research Institute, Universitat Autònoma de Barcelona, Badalona, Spain; ^16^UMI 233 TransVIHMI/INSERM1175, Institut de Recherche pour le Développement, University of Montpellier, Montpellier, France; ^17^Division of Clinical Microbiology, Department of Laboratory Medicine, Karolinska Institutet, Stockholm, Sweden; ^18^AIDS Reference Laboratory, Department of Microbiology and Infection Control, Universitair Ziekenhuis Brussel, Brussels, Belgium; ^19^Unidade de Microbiologia, Center for Global Health and Tropical Medicine, Instituto de Higiene e Medicina Tropical, Universidade Nova de Lisboa, Lisbon, Portugal; ^20^AIDS Reference Laboratory, Department of Microbiology, Saint-Pierre University Hospital, Brussels, Belgium; ^21^AIDS Reference Laboratory, University Hospitals Leuven, Leuven, Belgium; ^22^AIDS Reference Laboratory, Department of Clinical Chemistry, Microbiology and Immunology, Ghent University, Ghent, Belgium; ^23^Department of Biosystems Science and Engineering, ETH Zürich, Basel, Switzerland; ^24^Swiss Institute of Bioinformatics, Lausanne, Switzerland

**Keywords:** HIV-1, sub-subtype F1, Belgium, phylodynamics, men-having-sex-with-men, treatment as prevention

## Abstract

Human immunodeficiency virus type 1 (HIV-1) non-B subtype infections occurred in Belgium since the 1980s, mainly amongst migrants and heterosexuals, whereas subtype B predominated in men-having-sex-with-men (MSM). In the last decade, the diagnosis of F1 sub-subtype in particular has increased substantially, which prompted us to perform a detailed reconstruction of its epidemiological history. To this purpose, the Belgian AIDS Reference Laboratories collected HIV-1 *pol* sequences from all sub-subtype F1-infected patients for whom genotypic drug resistance testing was requested as part of routine clinical follow-up. This data was complemented with HIV-1 *pol* sequences from countries with a high burden of F1 infections or a potential role in the global origin of sub-subtype F1. The molecular epidemiology of the Belgian subtype F1 epidemic was investigated using Bayesian phylogenetic inference and transmission dynamics were characterized based on birth-death models. F1 sequences were retained from 297 patients diagnosed and linked to care in Belgium between 1988 and 2015. Phylogenetic inference indicated that among the 297 Belgian F1 sequences, 191 belonged to a monophyletic group that mainly contained sequences from people likely infected in Belgium (OR 26.67, 95% CI 9.59–74.15), diagnosed in Flanders (OR 7.28, 95% CI 4.23–12.53), diagnosed at a recent stage of infection (OR 7.19, 95% CI 2.88-17.95) or declared to be MSM (OR 34.8, 95% CI 16.0–75.6). Together with a Spanish clade, this Belgian clade was embedded in the genetic diversity of Brazilian subtype F1 strains and most probably emerged after one or only a few migration events from Brazil to the European continent before 2002. The origin of the Belgian outbreak was dated back to 2002 (95% higher posterior density 2000–2004) and birth-death models suggested that its extensive growth had been controlled (*R_e_* < 1) by 2012, coinciding with a time period where delay in antiretroviral treatment initiation substantially declined. In conclusion, phylogenetic reconstruction of the Belgian HIV-1 sub-subtype F1 epidemic illustrates the introduction and substantial dissemination of viral strains in a geographically restricted risk group that was most likely controlled by effective treatment as prevention.

## Introduction

Human immunodeficiency virus type 1 (HIV-1) originated after at least four separate cross-species transmission events from non-human primates to humans in central Africa, which resulted in four groups (M, N, O, and P) that emerged in the early twentieth century (Peeters et al., 2014). Since then, HIV-1 group M lineages have diversified and the current genetic diversity existing within the pandemic group M is the result of subsequent evolution and spread within the human population. Using phylogenetic analysis, HIV-1 group M can be classified into nine major subtypes (A–D, F–H, J, and K) and a few sub-subtypes (like F1 and F2) which reflects a combination of several founder effects and incomplete sampling of the original source population in central Africa (Rambaut et al., 2004). On the other hand, the various circulating recombinant forms (CRFs) originated from co-circulation of subtypes that previously evolved in compartmentalized epidemics.

The HIV-1 subtype B lineage that is responsible for the majority of HIV-1 infections in Europe, was first exported from central Africa to the Caribbean region around 1967 from where it was subsequently introduced in the United States and spread over to other American and European countries, mainly via networks of men-having-sex-with-men (MSM) and people who injects drugs (PWID) (Abecasis et al., 2013; Junqueira and Almeida, 2016; Magiorkinis et al., 2016; Worobey et al., 2016). HIV-1 subtype B was the predominant subtype among Belgians who were diagnosed with HIV-1 between 1985 and 1994 in Antwerp (Belgium), although the presence of other pure subtypes such as A, C, D, F, G, and H already revealed an early introduction of HIV-1 non-B subtypes to the local population (Fransen et al., 1996). Nevertheless, Belgian MSM remained almost exclusively infected with subtype B strains. These findings were confirmed in an independent study where heterosexual contact, African origin and more recent diagnosis were predictors for non-B infection in HIV-1 patients diagnosed between 1983 and 2001 in two other Belgian cities (Brussels and Leuven) (Snoeck et al., 2004). Subsequent local and national surveillance studies that included HIV-1 diagnoses between 1998 and 2012 confirmed the association between subtype B infections, MSM and Caucasian origin. In contrast, HIV-1 non-B infections in Belgium, of which HIV-1 subtype A, C, and CRF02_AG infections were the most prevalent, were associated to migrants from sub-Saharan Africa and individuals showing heterosexual risk behavior (Vercauteren et al., 2008; Chalmet et al., 2010; Pineda-Peña et al., 2014).

In the last decade, sub-subtype F1 diagnoses have markedly increased among newly diagnosed HIV-1 patients linked to care in Belgium. In the light of two Spanish studies that reported lower therapy response rates in HIV-1 sub-subtype F1 infections (Pernas et al., 2014; Cid-Silva et al., 2018), we aimed to investigate the evolutionary history and distinctive socio-demographic and epidemiological features of the HIV-1 sub-subtype F1 epidemic in Belgium. The spatiotemporal origin of the ancestor of the Belgian F1 epidemic and evolutionary links among locations were determined, risk factors were identified, and transmission dynamics were quantified.

## Materials and Methods

### Viral Sequences and Associated Information

Human immunodeficiency virus type 1 sub-subtype F1 *pol* sequences spanning the gene fragments that encode protease (PR) and the 5′-end of reverse transcriptase (RT) were collected from all patients for whom drug resistance testing was requested as part of routine clinical follow-up at six AIDS reference laboratories (ARL) in Antwerp, Brussels, Ghent, and Leuven (up until March 2015). These ARL perform approximately 90% of the HIV-1 drug resistance testing in Belgium. HIV-1 sub-subtype F1 *pol* sequences were requested from partners in other European and African countries where circulation of F1 infections has been reported (Palma et al., 2007; Paraschiv et al., 2009; Frentz et al., 2010; Lai et al., 2012; Ivanov et al., 2013; Sayan et al., 2013; Alexiev et al., 2015). Furthermore, all available HIV-1 sub-subtype F1 *pol* sequences were extracted from the Los Alamos HIV Sequence Database (LANL)^1^ (Triques et al., 1999; Montano et al., 2005; Aulicino et al., 2007). Most viral genetic sequences were annotated with sampling time and location, which were used as calibration and discrete traits, respectively, in an analysis of spatiotemporal dispersion patterns (Supplementary Table S1). Patient socio-demographics and detailed sample information were collected from mandatory reporting and medical records at the participating ARL in Belgium (gender, year of birth, HIV-1 diagnosis date, infection stage at diagnosis, country of birth, probable country of infection, sexual risk factor for HIV-1 acquisition, viral load, CD4 and CD8 count associated to the HIV-1 *pol* sequence, and therapy initiation date). Infection stage at diagnosis was defined based upon laboratory data: HIV-1 confirmation results with a positive p24 test combined with a negative or undetermined blot or INNO-LIA HIV I/II (Fujirebio Europe, Ghent, Belgium) were classified as acute infections while results with a positive blot lacking a p31 band as recent infections (Verhofstede et al., 2017). In all other instances, at least when records were available, infections were classified as chronic. The study was approved by the Ethical Committee Research UZ/KU Leuven (reference S58359). This study did not require written informed consent by study participants as it only used data collected within routine clinical practice, but patients who opted out of research studies were not included. The entire dataset for this manuscript is not publicly available because it represents a dense sample of our epidemic and inappropriate use could endanger the privacy of the patients. A proportional down-sampling of the dataset (30%) is accessible via GenBank (accession numbers, MK458435-MK458523). Requests to access the entire dataset combined with well-defined project proposals can be mailed to the corresponding author who will submit it to the local ethical committee for ethical and legal clearance when not in conflict with already on-going research.

### Quality Control and Compilation of Sequences

The quality of HIV-1 sequences that were generated by the Belgian ARL was assured by quality management systems that are conform to the ISO 15189 standard. In addition, the quality of all collected HIV-1 sequences was subsequently verified using the Quality Control tool hosted on the HIV sequence database LANL. Only sequences with no evidence of hyper-mutations and a maximum of 7 stop codons and/or frameshifts were retained for further analyses. Only pure sub-subtype F1 strains were retained in the study. Therefore, HIV-1 subtype classification was confirmed using the online automated subtyping tools COMET v1.0 (Struck et al., 2014) and Rega v3 (Pineda-Peña et al., 2013). In case of a non-F1 classification by COMET v1.0 or Rega v3 (7% discordant classifications), sequences were subsequently subjected to jpHMM (Schultz et al., 2009; version March 2015). If scored as F1 or F2 sub-subtype by jpHMM, recombination was ruled out using Simplot (Lole et al., 1999). Four HIV-1 subtype B reference sequences were added to the dataset, serving as an outgroup for subsequent phylogenetic analyses (GenBank accession numbers K03455, AY423387, AY173951, and AY331295). Nucleotide sequences were aligned using Muscle v3.7 (Edgar, 2004) and manually edited in MEGA 5 (Tamura et al., 2011). Nucleotide positions encoding for 43 surveillance drug resistance mutations (Bennett et al., 2009) were removed from the alignment to avoid the effect of drug-induced convergent evolution. Duplicate sequences were identified using the ElimDupes tool (LANL) and were removed when originating from the same sample. For patients with multiple sequences, only the first sequence was retained. The final alignment contained 1866 HIV-1 sub-subtype F1 sequences trimmed to the common length of 798 nucleotide positions, spanning sites 2253 to 2549 and 2661 to 3290 (as compared to the HIV-1 subtype B reference sequence HXB2, accession number K03455) (Supplementary Table S1).

### Phylogenetic and Phylogeographic Analyses

The spatiotemporal origin of the Belgian HIV-1 sub-subtype F1 epidemic was investigated using a Bayesian approach. Prior to this analysis, we examined the accumulation of divergence over the sampling time span using Tempest v1.5 (Rambaut et al., 2016) to assure that sampling dates could be used as calibration. Markov chain Monte Carlo analyses were performed using BEAST v1.8.4 in conjunction with BEAGLE to improve the computational performance (Ayres et al., 2012; Suchard et al., 2018). We specified a GTR nucleotide substitution model with discrete gamma distributed rate variation among sites. Among-lineage variation in evolutionary rates was modeled using a lognormal relaxed molecular clock and a flexible non-parametric Bayesian Skygrid was specified as a coalescent prior. The analyses were run in triplicate for 100 million states and trees were sampled every 10,000th state. Convergence and mixing properties of the Markov chain Monte Carlo chains were assessed with Tracer v1.6 (Rambaut et al., 2018). Results of several chains were combined after removal of the burn-in, and a MCC tree was summarized using TreeAnnotator within the BEAST package. The timed migration process was reconstructed using a model that allows for non-reversible migration rates. Trees were visualized with FigTree v1.4.3 and iTOL v4.2.3 (Letunic and Bork, 2016).

### Phylodynamic Analysis of the Infection Cluster Including Belgian Sequences

Phylodynamic analyses were performed on an infection cluster that mainly contained sequences from our Belgian cohort. A birth-death model was applied to the Belgian sequences included in this infection cluster (191 sequences) in order to quantify transmission dynamics through time. The analyses were performed in BEAST v2 (Bouckaert et al., 2014) using the BDSKY add-on (Stadler et al., 2013). The molecular evolution and clock model were chosen as in the phylogeographic analysis. For the birth-death-skyline parameters, we used a LogNorm(0,1) prior for the effective reproductive number, a LogNorm(0,1) prior for the becoming-non-infectious rate in units per year (i.e., the inverse of the time duration of being infectious in unit of years), and a Unif(0,infinity) prior for the origin of the epidemic. The dimension of the effective reproductive number and the becoming-non-infectious rate was set to 10, meaning the parameters were estimated for 10 equally spaced intervals between the origin and the most recent sample. The sampling proportion was fixed to 0.65 (and 0.8 in a second analysis) for the time between the first and last sample, and 0 prior to the first sample.

### Statistical Analysis

Socio-demographic, virological and clinical variables were reported as absolute numbers, percentages and medians with their respective interquartile ranges (IQR), where appropriate. Their association with a Belgian F1 outbreak was evaluated using univariate and multivariate logistic regression in the statistical R software v3.5.1, with statistical significance set at *p*-value < 0.05. For these analyses, only patients with available information for a specific variable were included.

## Results

### Increase of Subtype F1 Diagnoses in Belgium

**FIGURE 1:**
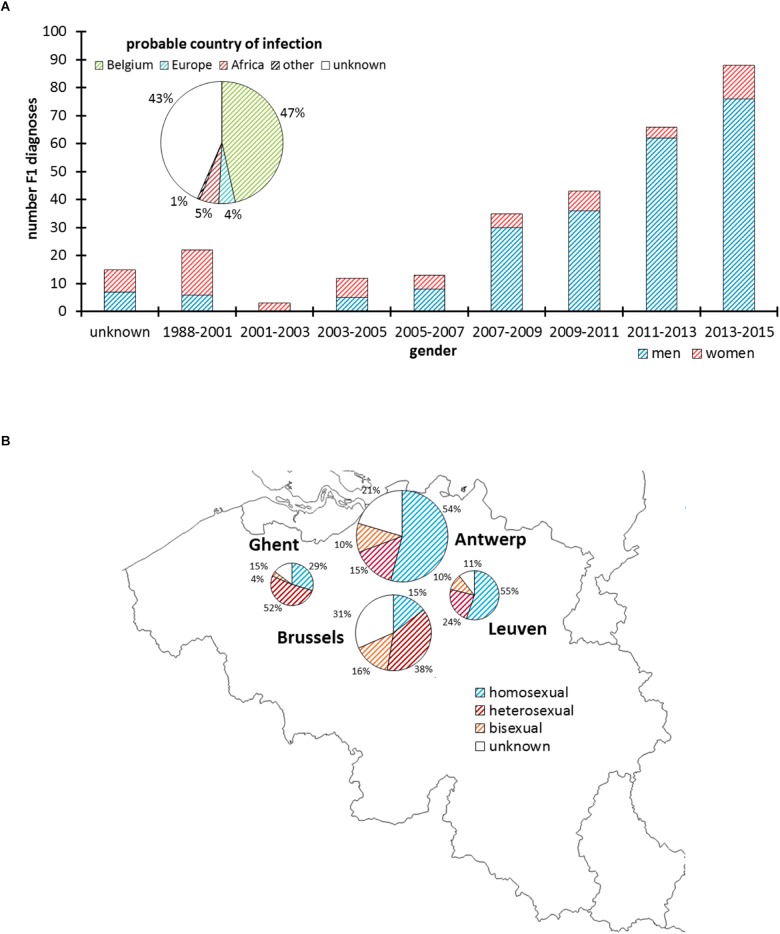
HIV-1 sub-subtype F1 diagnoses in Belgium. Panel **(A)** Number of men and women diagnosed with an HIV-1 sub-subtype F1 infection according to 2-year intervals and based upon a *pol*-genotypic drug resistance test that was requested as part of routine clinical follow-up. The pie chart displays the proportion of people who probably acquired the HIV-1 sub-subtype F1 infection in Belgium, Europe, Africa, or another or unknown region. Panel **(B)** Geographic and proportional distribution of HIV-1 sub-subtype F1 diagnoses in the AIDS Reference Laboratories of Antwerp, Brussels, Leuven, and Ghent. The pie charts display the proportion of people reporting homosexual, heterosexual, or bisexual contacts as risk factor for HIV-1 infection.

Up to February 2015, 297 HIV-1 sub-subtype F1 infections were newly diagnosed in the participating Belgian ARL (Figure 1), of which 137 were analyzed in Antwerp (46%), 95 in Brussels (32%), 38 in Leuven (13%), and 27 in Ghent (9%). The median age of people diagnosed with an HIV-1 sub-subtype F1 infection was 34 years (IQR 29–41) and 77% were men. Over the course of 15 years, the number of HIV-1 sub-subtype F1 diagnoses increased from 3 in March 2001 – February 2003 to 88 in March 2013 – February 2015 (Figure 1A). Sub-subtype F1 infections were initially mainly diagnosed in women (65%), but after February 2005 men became the most affected gender (87%). Of the sub-subtype F1 infections for which the respective information was registered, the majority were diagnosed in people born in Belgium (59%, 141/238) and in people who declared that they probably acquired their infection in Belgium (82%, 138/168). The sub-subtype F1 infections were found in homosexual (51%, 117/231), heterosexual (35%, 80/231), and bisexual risk (15%, 34/231) groups (Figure 1B).

### The Belgian Sub-Subtype F1 Epidemic Within a Global Context

Bayesian phylogenetic analyses were used to estimate the geographical and temporal origin of the HIV-1 sub-subtype F1 strains in our national cohort and to investigate viral dissemination from Belgium to other countries. The final dataset included 1866 sequences that were mainly sampled in Europe (75%) and Brazil (20%) (Supplementary Table S1). We found sufficient temporal signal to pursue phylodynamic analysis and used the resulting empirical distribution of the dated phylogenetic trees in a subsequently phylogeographic analysis.

**FIGURE 2:**
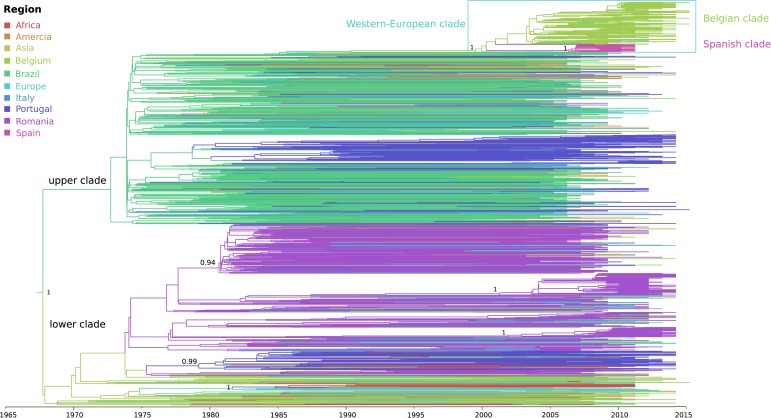
Phylogeographic reconstruction of the Belgian HIV-1 sub-subtype F1 epidemic. The branches in the time-scaled maximum clade credibility tree constructed from the complete dataset of 1866 *pol* sequences are colored by their most probable location state (see legend), which could be a specific sampling country or grouped as a continent when less than 30 sequences were available for a specific country (Supplementary Table S1). The HIV-1 sub-subtype F1 strains that are associated with the local outbreak (191 lineages), most probably entered Belgium via Latin-America, more particularly Brazil, a transmission event that is estimated to have occurred around 2002 (95% HPD 2000 – 2004). The 106 Belgian sequences that do not belong to the monophyletic group, are dispersed within less recent clades, grouping with lineages from Africa, Latin-America and Europe. Posterior root node support is visualized in a selection of nodes.

The phylogeographic analysis revealed the divergence of sub-subtype F1 into two well-supported clades for which (i) one clade included mainly Brazilian and Portuguese strains together with a Belgian subclade, while (ii) the other predominantly Belgian, Romanian and Portuguese sequences (upper and lower clade in Figure 2, Supplementary Figure S1). Their divergence dates back 50 years ago (1968, 95% higher posterior density (HPD) interval of 1963–1973), with an estimated start of local dissemination of HIV-1 F1 in Brazil around 1973 (95% HPD 1969–1975) and subsequently in Portugal around 1979 (95% HPD 1976–1981) (Figure 2 upper clade). In this upper clade (i), one large Belgian cluster, subsequently referred to as the sub-subtype F1 Belgian outbreak, is embedded within a Western-European clade that in turn is nested within the larger Brazilian clade. The Belgian clade contains four sequences sampled outside Belgium (one from Brazil, one from Luxembourg, and two from Germany), while the Western-European clade consists of the Belgian clade together with three basal sequences from Switzerland, one large well-supported Spanish clade and one Romanian sequence that branches outside the Spanish and Belgian clade. The founder of the HIV-1 sub-subtype F1 strains that are currently circulating in Western-Europe, probably migrated from Brazil to Europe between 1990 and 1999 (95% HPD 1987–2002), whereas the origin of the Belgian and Spanish outbreaks were dated back to 2002 (95% HPD 2000–2004) and 2006 (95% HPD 2004–2008), respectively. Brazil is estimated as the most likely geographical origin of the Western-European clade because the Western-European clade was embedded within the large Brazilian sub-subtype F1 diversity and because one strain that was sampled in Brazil and three strains that were sampled from Brazilians residing in Belgium were also positioned within the Belgian clade. Although Belgium was assigned as ancestral location state of this Western-European clade, the long interconnecting branch suggests that current sampling is insufficient to provide conclusive results as to the specific migration event(s) that finally led to the two large outbreaks in Western Europe (Belgium and Spain).

**FIGURE 3:**
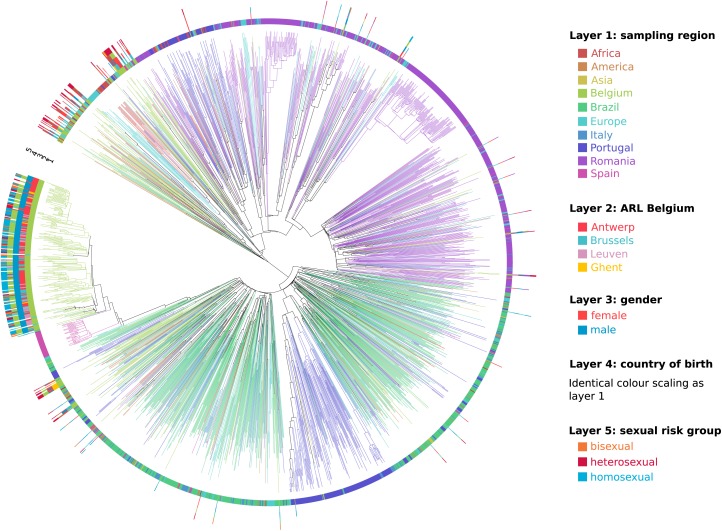
Circular representation of the maximum clade credibility tree of the Belgian sub-subtype F1 epidemic within a global context. Branches in the tree are colored according to sampling region (identical coloring scaling as in Figure 2), which is also displayed in layer 1. For the HIV-1 sub-subtype F1 strains that were sampled at the AIDS reference laboratories (ARL) in Belgium, the location of testing was displayed in layer 2, gender in layer 3, country of birth in layer 4, and sexual risk group in layer 5. This detailed information was largely lacking for the sequences sampled outside Belgium.

The remaining sub-subtype F1 sequences from our Belgian cohort represent single lineages or small clusters that are dispersed across the tree and are intermixed among viral lineages from Africa, Latin-America and Europe (Figure 2, 3). Several other strains from Sub-Saharan migrants residing in Belgium but who originated from the Democratic Republic of Congo (DRC), Congo, and Angola occupied the most basal positions of the tree (Figure 2 lower clade, Figure 3). They cluster with African lineages that probably migrated to Europe between 1972 and 1977 (95% HPD 1969–1980) and that were at the origin of several larger sub-subtype F1 outbreaks in Europe, more specifically in Portugal (1979, 95% HPD 1976–1982), Romania (1981, 95% HPD 1978–1983; 2001, 95% HPD 1996–2006; and 2002, 95% HPD 1998–2006), and Bulgaria (1982, HPD 1979–1985). As, we defined a location trait according to country of sampling in our analysis, the over-representation of Belgian sequences – probably to a large extent reflecting African diversity – led Belgium to be inappropriately assigned as the ancestor location of this lower clade that contained many African, Romanian, Portuguese, and other European lineages (Figure 2 lower clade).

### Socio-Epidemiological Correlates With the Sub-Subtype F1 Outbreak in Belgium

As one or only a few sub-subtype F1 lineages have successfully established local transmissions among 191 of 297 people diagnosed with a sub-subtype F1 infection in Belgium (64%), we used univariate and multivariate logistic regression to identify socio-epidemiological variables that were associated with this successful sub-subtype F1 outbreak in Belgium (Table 1). HIV-1 patients who were diagnosed with a sub-subtype F1 infection at the AIDS reference laboratories in Antwerp (OR 6.80, 95% CI 3.86–11.99), Leuven (OR 4.22, 95% CI 1.59–11.16) or more broadly in the region of Flanders (OR 7.28, 95% CI 4.23–12.53) had significantly higher odds of belonging to this F1 outbreak, compared to people who were diagnosed with a sub-subtype F1 infection elsewhere in Belgium. In addition, many other variables of the sub-subtype F1 infections diagnosed in Belgium displayed significantly higher odds of being part of the outbreak, of which the most informative were male gender (OR 52.42, 95% CI 19.90–138.08), infection in MSM (combining homosexuals and bisexuals) (OR 34.8, 95% CI 16.0–75.6), probable infection in Belgium (OR 26.67, 95% CI 9.59–74.15), born in Belgium (OR 12.10, 95% CI 6.35-23.08), recent infection stage at diagnosis (OR 7.19, 95% CI 2.88–17.95) and higher viral load (OR 2.36, 95% CI 1.78–3.13). In multivariate analysis, sub-subtype F1 infections in MSM and in people born in Belgium remained to display a higher likelihood for belonging to the sub-subtype F1 Belgian outbreak [OR 7.82, 95% CI 3.80–16.11 and OR 2.01 (1.06–3.78), respectively]. The variables sampling region, ARL in Belgium, gender, country of birth and sexual risk group were visualized for all Belgian sequences in a circular representation of the phylogenetic tree (Figure 3).

**Table 1:** Characteristics of people diagnosed with an HIV-1 sub-subtype F1 infection in Belgium and factors associated with this local outbreak.

Characteristic	Local outbreak (*n* = 191)	Other (*n* = 106)	Univariate logistic regression		Multivariate logistic regression
				
			OR (95% CI)	*p*	OR (95% CI)	*p*
AIDS Reference Laboratory [% (*n*)]						
Antwerp	61.3 (117)	18.9 (20)	6.80 (3.86–11.99)	< 0.0001		
Brussels	16.7 (32)	59.4 (63)	0.14 (0.08–0.24)	< 0.0001		
Leuven	17.3 (33)	4.7 (5)	4.22 (1.59–11.16)	0.0037		
Ghent	4.7 (9)	17.0 (18)	0.24 (0.10–0.56)	0.0009		
Region [% (*n*)]						
Flanders	83.3 (159)	40.6 (43)	7.28 (4.23–12.53)	< 0.0001		
Gender [% (*n*)]						
Male	97.4 (186)	41.5 (44)	52.42 (19.90–138.08)	< 0.0001		
Age at diagnosis [years]						
Median (IQR)	34 [29, 41] (190)	34 [27, 40] (92)	1.03 (1.00–1.06)	0.0343		
Diagnosis date [dd-mm-yyyy]						
Median [IQR] (*n*)	09-05-2012	24-1-2008	1.33 (1.23–1.45)	< 0.0001		
	[21-03-2010, 23-11-2013] (190)	[3-4-2002, 31-10-2011] (92)				
Infection stage at diagnosis [% (*n*)]						
Acute	10.1 (16/158)	2.2 (1/45)	4.96 (0.64–38.45)	0.1256		
Recent	52.5 (83/158)	13.3 (6/45)	7.19 (2.88–17.95)	< 0.0001		
Chronic	37.4 (59/158)	84.5 (38/45)	0.11 (0.05–0.26)	< 0.0001		
Unknown	17.2 (33)	57.5 (61)				
Country of birth [% (*n*)]						
Belgium	77.8 (123/158)	22.5 (18/80)	12.10 (6.35–23.08)	< 0.0001	2.01 (1.06–	0.0316
Other	22.2 (35/158)	77.5 (62/80)			3.78)	
Unknown	17.2 (33)	24.5 (26)				
Probable country of infection [% (*n*)]						
Belgium	95.2 (120/126)	42.9 (18/42)	26.67 (9.59–74.15)	< 0.0001		
Other	4.8 (6/126)	57.1 (24/42)				
Unknown	34.0 (65)	60.4 (64)				
Sexual risk group [% (*n*)]						
Homosexual contacts	69.2 (110/159)	9.7 (7/72)	20.85 (8.92–48.73)	< 0.0001		< 0.0001
Bisexual contacts	18.2 (29/159)	7.0 (5/72)	2.99 (1.11–8.08)			
Heterosexual contacts	12.6 (20/159)	83.3 (60/72)	0.03 (0.01–0.06)	0.0308		
Men-having-sex-with-men	87.4 (139/159)	16.7 (12/72)	34.8 (16.0–75.6)	< 0.0001	7.82 (3.80–	
Unknown	16.8 (32)	32.1 (34)		< 0.0001	16.11)	
Viral load at sequencing [log_10_						
copies/ml] Median [IQR] (*n*)	5.18 [4.61–5.79] (191)	4.36 [3.75, 4.92] (101)	2.36 (1.78–3.13)	< 0.0001		
CD4+ at sequencing [cells/μl]						
Median [IQR] (n)	488 [358–645] (184)	334 [175, 528] (89)	1.00 (1.00–1.00)	< 0.0001		
CD8+ at sequencing [cells/μl]						
Median [IQR] (*n*)	1059 [697–1598] (173)	1041 [778, 1390] (67)	1.00 (1.00–1.00)	0.2980		


*A univariate and multivariate logistic regression analysis was performed to identify socio-epidemiological variables that were associated with the local HIV-1 F1 outbreak in Belgium (*n* = 191). A *p*-value of < 0.05 was considered to be significant. Abbreviations: n, number, IQR, interquartile range, OR, odds ratio, CI, confidence interval.*

### Reduced Onward Transmission of Sub-Subtype F1 Strains Coincident With an Earlier Initiation of Antiretroviral Therapy

**FIGURE 4:**
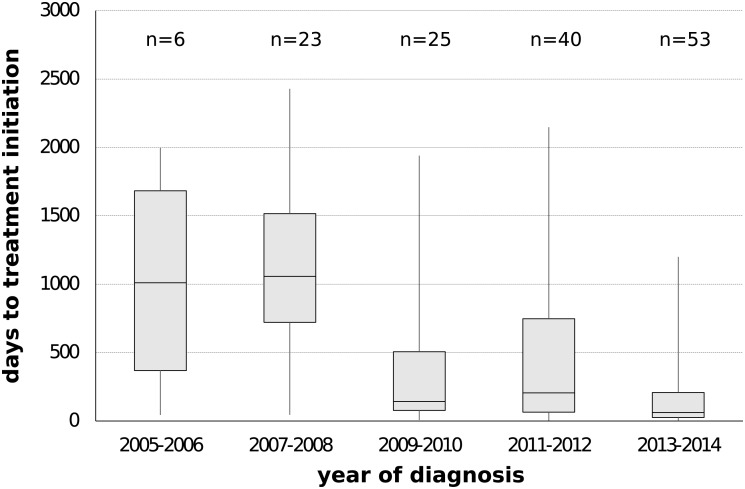
Delay in antiretroviral treatment among people infected with sub-subtype F1 strains belonging to the local outbreak in Belgium. The number of days between diagnosis date and treatment initiation date were plotted according to 2-year intervals of diagnosis date. Information on prescribed antiviral therapy and diagnosis date was available for 77% of the HIV-1 patients belonging to the local outbreak in Belgium (147/191).

**FIGURE 5:**
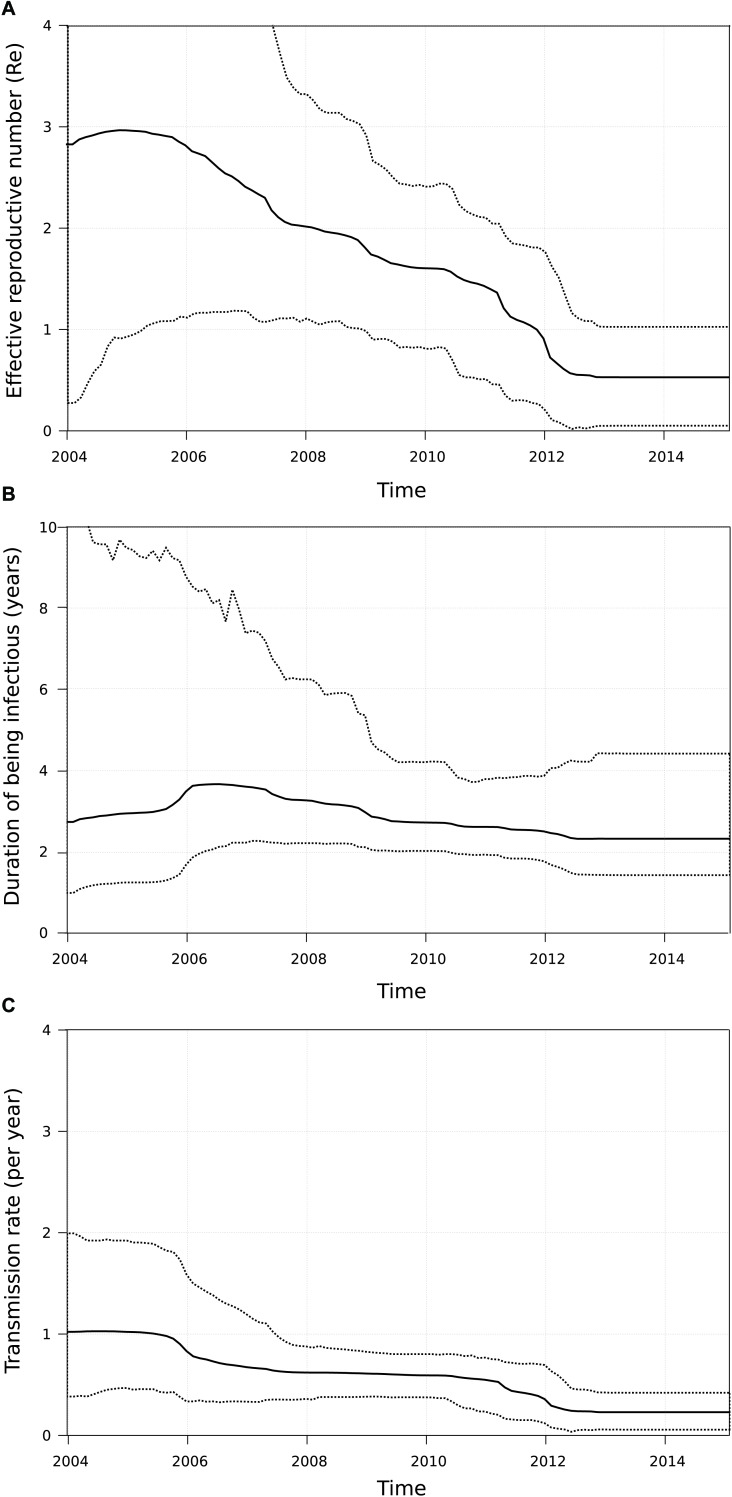
Birth-death model applied to the sub-subtype F1 outbreak in Belgium. For all patients belonging to the large Belgian cluster (n = 191), the number of secondary infections per infected person (effective reproductive number Re, panel **(A)**), the median infectious time in years (panel **(B)**) and the number of secondary infections per infected person per year (transmission rate, panel **(C)**) was estimated. After the onset of the outbreak both Re and transmission rate started to decline, with the largest drop occurring after 2011.

Since the onset of the sub-subtype F1 outbreak in Belgium (2002, 95% HPD 2000–2004), we covered a time period where antiretroviral therapy was initially still deferred in asymptomatic patients with CD4 count above 350 cells/mm^3^ (European AIDS Clinical Society, 2005). This general practice gradually switched to the consideration of antiretroviral therapy for asymptomatic patients with CD4 counts between 350 and 500 cells/mm^3^ (European AIDS Clinical Society, 2007) and above 500 cells/mm^3^ (European AIDS Clinical Society, 2013). Given the benefits of antiretroviral therapy in reducing infectivity, we wanted to quantify transmission dynamics through time using a birth-death model and monitor the trends in therapy uptake in our specific setting. Therapy and diagnosis information was available for 147 HIV-1 patients whose viral strains belonged to the sub-subtype F1 outbreak in Belgium (77%, 147/191). The delay in therapy initiation decreased from a median of 1011 days (IQR 368–1683) or almost 3 years for patients diagnosed in 2005 and 2006 to a median of 61 days (IQR 26–208) or about 2 months for patients diagnosed in 2013 and 2014 (Figure 4). In the birth-death model, assuming a constant 65% sampling proportion, the number of expected secondary infections per infected person (effective reproductive number R_e_) was estimated to be a median of 3 in 2005 and gradually declined to a median of 1.5 in 2011, after which a substantial drop to below 1 was observed (Figure 5). During this time window, the median infectious time also declined and the number of expected secondary infections per infected person and per year (transmission rate) decreased from a median of 1 to well below 1. Sensitivity analysis using a constant sampling proportion of 80%, as the diagnosis and sequencing rate is expected to be higher in MSM, showed similar results and suggests that the estimates are relatively robust to prior specification on the sampling proportion.

## Discussion

Human immunodeficiency virus type 1 subtype F most likely emerged from the early 20th century HIV-1 diversity in the DRC, where its current prevalence remains less than 5% (Vidal et al., 2000; Faria et al., 2014; Rodgers et al., 2017). This subtype accounts for less than 1% of HIV-1 infections worldwide and is subdivided into two sub-subtypes F1 and F2, of which the detection of the latter remains mostly restricted to Cameroon (Brennan et al., 2008; Hemelaar et al., 2011). In contrast, sub-subtype F1 strains have spread to other Central African countries, reaching a particularly high prevalence of 14% in Angola (Afonso et al., 2012). In Belgium, the burden of sub-subtype F1 infections among newly diagnosed HIV-1 patients increased to 11.1% in 2015. The aim of this study was to characterize the epidemic dynamics of this non-B subtype epidemic in Belgium by using phylogenetic approaches and the analysis of socio-epidemiological variables.

### The Dissemination of HIV-1 Sub-Subtype F1 Across the World

Our and other phylogenetic analyses confirmed that the most basal F1 sequences were isolated from people originating from Central African countries and that the major migration events out of Africa occurred in the 1970s and early 1980s seeding some larger F1 epidemics over the world (Mehta et al., 2011; Bello et al., 2012; Lai et al., 2014). During the Angolan civil war that started in 1975 and lasted for more than 16 years, an extensive migration of troops, settlers and refugees was observed between Angola, DRC, Portugal, Brazil, Cuba, Russia, and several Eastern-European countries and this is considered as an important factor in the global dissemination of F1. In Brazil, sub-subtype F1 infections have reached prevalence rates of more than 7% and the strains are currently circulating among various risk groups (Brindeiro et al., 2003). It is the most probable epicenter of F1-related epidemics within the Southern Cone and one of the sources of the Portuguese sub-subtype F1 epidemic (Aulicino et al., 2007; Bello et al., 2007, 2012; Carvalho et al., 2015). Unrelated to the Brazilian epidemic, sub-subtype F1 was initially introduced and spread among adult heterosexuals in Romania. In this Eastern-European country, it subsequently reached a very high prevalence rate of more than 70% due to several major outbreaks among children in orphanages and people using intravenous drugs (Dumitrescu et al., 1994; Bandea et al., 1995; Apetrei et al., 1998; Op De Coul et al., 2000; Guimarães et al., 2009; Paraschiv et al., 2009; Mehta et al., 2011; Mbisa et al., 2012; Niculescu et al., 2014). Both Brazil and Romania were at the origin of the F1 epidemic in Italy, where Brazilian strains contributed to many more introduction events characterized with a more extensive spread (Lai et al., 2014). Since then, the prevalence of sub-subtype F1 increased gradually among Italian descendants and mainly among self-declared heterosexual men, although phylogenetic analyses suggested that some of these self-declared heterosexual men might have been infected through homosexual contact (Bracciale et al., 2009;Lai et al., 2012, 2014).

Although our and a previous study indicated that another sub-subtype F1 lineage migrated from Brazil to Europe during the 1990s, this lineage obtained only more recently epidemic proportions in two countries, respectively, about 16 years ago in Belgium and 12 years ago in Spain (Paraskevis et al., 2015). In both instances, F1 was introduced in local MSM networks, a factor probably important in its subsequent spread and circulation at high rates (Thomson et al., 2012). Our analyses did not indicate any intermixing between both epidemics and only sporadic and putative exports from Belgium to Luxembourg (one) and Germany (two) were detected. This is in agreement with another study that traced the HIV-1 subtype B mobility in Europe and that characterized Belgium primarily as a sink of HIV-1 lineages rather than a source (Paraskevis et al., 2009). Although, we confirmed previous findings on the global spread of HIV-1 sub-subtype F1, our sampling approach might not have been sensitive enough to capture the specific migration events that resulted into these two large outbreaks or into extensive onward transmission to other countries. For our analyses, we collected HIV-1 *pol* sequences from countries with a known high burden of F1 infections or a potential role in the global spread of sub-subtype F1. In this dataset, Belgium, Romania, Portugal, Luxembourg, and Bulgaria were the best-represented countries as they contributed a dense sampling, whereas Italy provided a down-sampling proportional to their local sub-subtype F1 diversity. However, other countries might have been undersampled as they were represented by sequences that were received from single cohorts or by sequences that were collected from public databases. Such sample biases call for caution in interpreting the results of phylogeographic estimates.

### The Belgian HIV-1 Sub-Subtype F1 Outbreak

The first HIV-1 sub-subtype F1 infections within the local outbreak cluster were diagnosed in 2005. It concerned one chronic infection in Leuven and one infection with unknown status in Antwerp, suggesting a time of about 3 years between the estimated introduction in Belgium (2002, 95% HPD 2000–2004) and the first case reported by the Belgian ARL. Although there is universal access to HIV-1 health care in Belgium and we included all patients for whom genotypic drug resistance testing indicated a sub-subtype F1 infection, whether at baseline or at therapeutic failure, our sequencing efforts in routine practice only cover approximately 60% of all newly HIV-1 diagnoses registered in the national database. However, we believe that we do capture the majority of patients who are contributing to the local spread, as sub-subtype F1 is mainly circulating among MSM, a group who is well engaged in medical care (Van Beckhoven et al., 2015). Any HIV-1 patient who seeks medical care in Belgium will receive a confirmation of his/her infection and will therefore be registered in the national database. Therefore, we believe that a large proportion of the people living with HIV-1 in Belgium who do not receive a baseline resistance test at diagnosis are people with an infection that was already acquired and diagnosed abroad and who are already on successful antiretroviral treatment upon arrival in Belgium, or these people are only temporarily in the country and do not enter the HIV-1 health care system in Belgium.

With the dense sampling of our local epidemic at hand, we aimed to estimate the effective reproductive number R_e_, the transmission (birth) rates and the time of being infectious in our viral population using birth-death modeling in order to gain insights into the dynamics of the outbreak. The initial high *R_e_*-value, high transmission rate and long times of being infectious were in agreement with the explosive increase of sub-subtype F1 infections in our national cohort. However, the mean *R_e_*-value declined substantially from 2012 onward and this event was associated with lower transmission rates and shorter durations of being infectious. These observations may be explained by the policy changes around that time when therapy eligibility thresholds for asymptomatic HIV-1 patients became less strict in Belgium. Indeed, medical records showed a remarkable dent in the median treatment delay among people belonging to our outbreak around 2009–2010. Although this relationship does not demonstrate causality, it is supported by studies where viral suppression by antiretroviral therapy has shown to be effective in preventing HIV-1 transmission among serodiscordant heterosexual and MSM couples (Cohen et al., 2011; Rodger et al., 2016; Bavinton et al., 2018). However, the relevant changes in transmission dynamics were not yet reflected in the number of new sub-subtype F1 diagnoses up to the start of 2015. This could suggest that the effects were too recent to be fully captured by our surveillance data of new diagnoses or that the conditions were not yet fully optimal to achieve the full potential of treatment as prevention in this outbreak (Okano et al., 2016). Although a substantial proportion of our outbreak was diagnosed at an acute and recent stage of infection, we should aim to increase testing and diagnosis rates among people at risk of acquiring HIV-1 and treat them without any delay. In this respect, the INSIGHT START study resolved the last concerns for immediate initiation of antiretroviral therapy in asymptomatic HIV-1 infections as this study showed that this strategy was superior to deferral until the CD4 count declined to 350 cells/mm^3^ and that it was not associated with an increased rate of adverse events (INSIGHT START Study Group et al., 2015).

In conclusion, this study revealed that sub-subtype F1 strains were introduced in Belgium around 2002 and that during a decade they spread exponentially among MSM who were mainly from Belgian descent, residing in Flanders and diagnosed at a recent stage of infection. Our birth-death phylodynamic estimates are in line with the expectations of the effectiveness of treatment as prevention because we identify a decline in the onward transmission of sub-subtype F1 strains that coincides with the implementation of earlier initiation of antiretroviral therapy in our cohort. Further follow-up is planned, especially within the context of the roll-out of antiretroviral therapy in early asymptomatic HIV-1 infections and of the implementation of pre-exposure prophylaxis which is reimbursed to people who are at increased risk for HIV-1 infection since June 2017.

## Data Availability

The datasets generated for this study can be found in GenBank, MK458435-MK458523.

## Author Contributions

KVL conceived and designed the study. LV, KF, IA, CB, LD, CS-D, SGR, PG, FI, RK, JR, MS, SP, RP, MP, AS, EV, SVW, MVR, CV, A-MV, and KVL collected and analyzed the epidemiological and viral data. LV, LC, TS, and PL performed the phylogenetic analyses and inferences. LV, LC, and KVL wrote the first draft of the manuscript. All authors contributed to manuscript revision, read and approved the submitted version.

## Conflict of Interest Statement

The authors declare that the research was conducted in the absence of any commercial or financial relationships that could be construed as a potential conflict of interest.

## Footnotes

1 http://www.hiv.lanl.gov
