# Supplementary material for: Earlier Initiation of Antiretroviral Treatment Coincides With an Initial Control of the HIV-1 Sub-Subtype F1 Outbreak Among Men-Having-Sex-With-Men in Flanders, Belgium
Source: Front Microbiol. 2019 Mar 26;10:613. doi: 10.3389/fmicb.2019.00613 (PMC6443750; doi:10.3389/fmicb.2019.00613)
Supplement: Supplementary file 1 [file Table_1.DOCX]

Table S1: HIV-1 sub-subtype F1 *pol* sequences included in phylogenetic analyses.

| **Sampling** | |  |  | **Sub-subtype F1 sequences (#)** | | |
| --- | --- | --- | --- | --- | --- | --- |
| **Continent** (% dataset) | **Country** (% dataset) | **Time-referenced** |  | **Partners** | **LANL** | **Total** |
| Europe (75%) | Romania (26%) | 100% |  | 302 | 183 | 485 |
|  | Belgium (16%) | 100% |  | 297 | 7 | 304 |
|  | Portugal (14%) | 99% |  | 246 | 15 | 261 |
|  | Italy (6%) | 93% |  | 77 | 44 | 121 |
|  | Spain (4%) | 96% |  | 4 | 63 | 67 |
|  | Germany (1%) | 92% |  | 23 | 3 | 26 |
|  | Luxembourg (1%) | 100% |  | 24 | 1 | 25 |
|  | United Kingdom (1%) | 0% |  | - | 23 | 23 |
|  | Bulgaria (1%) | 100% |  | 17 | 1 | 18 |
|  | Sweden (<1%) | 100% |  | 16 | 1 | 17 |
|  | Turkey (<1%) | 100% |  | 15 | - | 15 |
|  | Switzerland (<1%) | 100% |  | - | 6 | 6 |
|  | Other (1%) | 89% |  | - | 27 | 27 |
| Africa (2%) | Cape Verde (<1%) | 100% |  | - | 13 | 13 |
|  | Angola (<1%) | 100% |  | - | 12 | 12 |
|  | Democratic Republic of Congo (<1%) | 100% |  | 6 | 1 | 7 |
|  | Other (<1%) | 86% |  | 2 | 5 | 7 |
| America (22%) | Brazil (20%) | 86% |  | - | 375 | 375 |
|  | Other (2%) | 90% |  | - | 41 | 41 |
| Asia (<1%) |  | 100% |  | - | 13 | 13 |
| Unknown (<1%) |  | 100% |  | 3 | - | 3 |
| **Geo-referenced** | **> 99%** | **95%** |  | **1032** | **834** | **1866** |

LANL, Los Alamos HIV Sequence Database. More than 99% of the sequences were geo-referenced and 95% were time-referenced. For more than 95% of the sequences both sampling time and location were known.
